# Supplementary figures and images for: The Pseudomonas aeruginosa T3SS can contribute to traversal of an in situ epithelial multilayer independently of the T3SS needle
Source: mBio. 2025 Mar 14;16(4):e00266-25. doi: 10.1128/mbio.00266-25 (PMC11980567; doi:10.1128/mbio.00266-25)

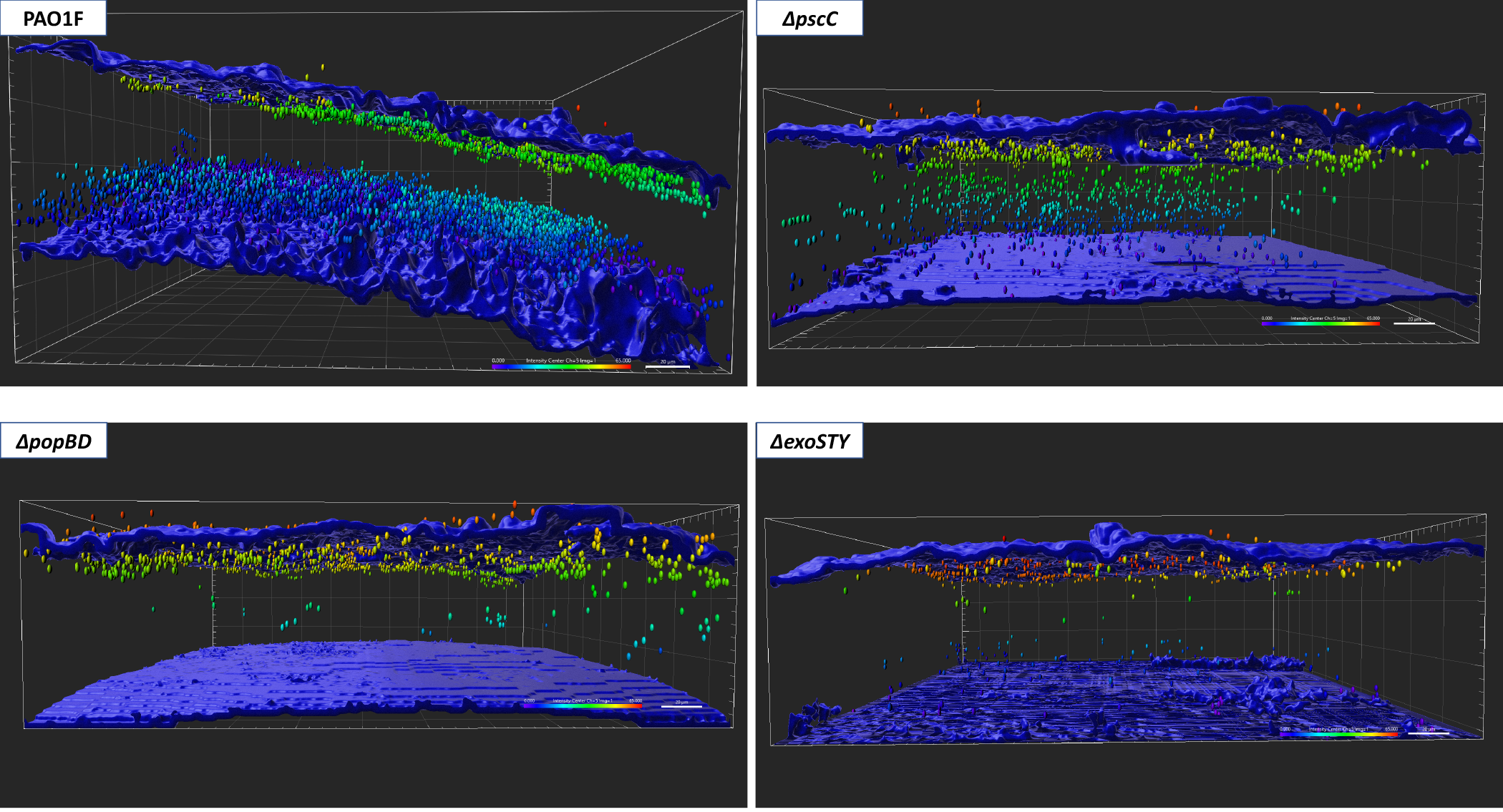

Supplement: Figure S1 — Representative images of corneal epithelium traversal by wild-type PAO1F compared to the ΔpscC, ΔpopBD, and ΔexoSTY mutants [file mbio.00266-25-s0001.tif]

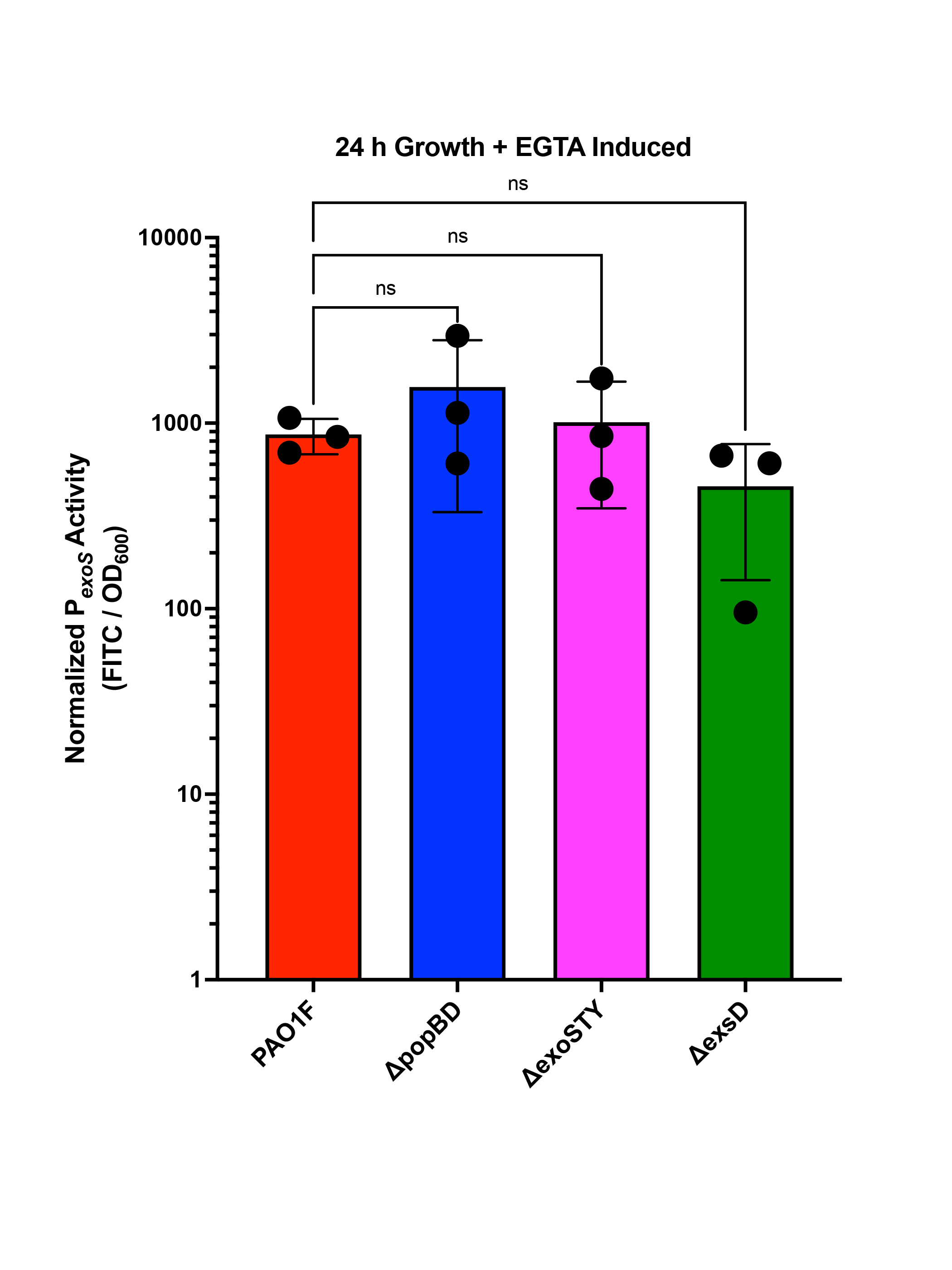

Supplement: Figure S2 — EGTA induction of P. aeruginosa T3SS gene expression was similar between PAO1 and T3SS mutants. [file mbio.00266-25-s0002.tif]

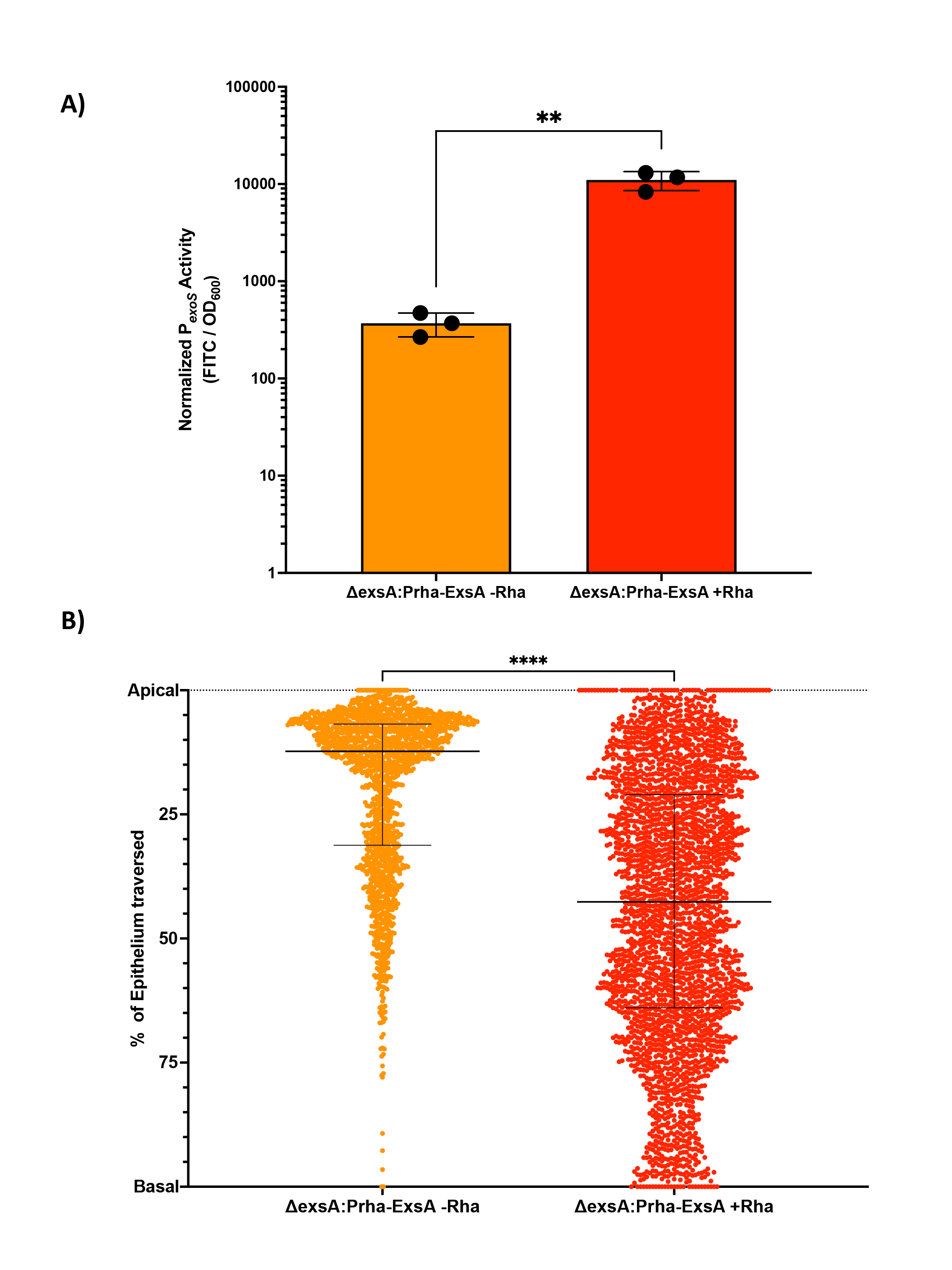

Supplement: Figure S3 — Rhamnose-induction of ExsA rescues ex vivo traversal. [file mbio.00266-25-s0003.tif]
