# Supplementary material for: The Pseudomonas aeruginosa T3SS can contribute to traversal of an in situ epithelial multilayer independently of the T3SS needle
Source: mBio. 2025 Mar 14;16(4):e00266-25. doi: 10.1128/mbio.00266-25 (PMC11980567; doi:10.1128/mbio.00266-25)
Supplement: Supplemental Material Legends — Legends for Fig. S1 to S3. [file mbio.00266-25-s0004.docx]

**The *Pseudomonas aeruginosa* T3SS can contribute to traversal of an *in situ* epithelial multilayer independently of the T3SS needle. Jedel *et al*. Supplemental Material**

**Supplemental Figure S1. Comparison of corneal epithelium traversal by *P. aeruginosa* and its T3SS mutants.** Representative images of corneal epithelium traversal by wild-type PAO1F compared to the Δ*pscC*, Δ*popBD* and Δ*exoSTY* mutants color-coded for traversal depth.

**Supplemental Figure S2. EGTA induction of *P. aeruginosa* T3SS gene expression was similar between PAO1 and T3SS mutants.** OD_600_-normalized GFP signal from pJNE05 (P*exoS*) carried by PAO1F or its T3SS mutants in the translocon pore Δ*popBD*, known exotoxins Δ*exoSTY*, or the transcriptional repressor Δ*exsD* after 24 h growth in T3SS-induction media [TSB plus gentamicin (200 μg/ml), monosodium glutamate (100 mM), glycerol (1 %) and EGTA (2 mM)]. Similar P*exoS* induction, i.e. T3SS induction, was observed across wild-type and mutants. N = 3 separate growth curves per group. Mean +/- standard deviation, ns = not significant (One-way ANOVA with Dunnett’s Multiple Comparisons).

**Supplemental Figure S3. Rhamnose-induction of ExsA rescues *ex vivo* traversal.** (A) OD_600_-normalized GFP signal from pJNE05 (P*exoS*) carried by a Δ*exsA* mutant complemented with rhamnose-inducible *exsA* with or without inclusion of rhamnose (Rha) (2 %) in the growth medium [TSB with gentamicin (200 μg/ml), monosodium glutamate (100 mM) and glycerol (1%)] after 24 h of growth. N = 3 separate growth curves per group. Mean +/- standard deviation, ** P < 0.01 (Paired Student’s t-Test). (B) Traversal depth after *ex vivo* infection of Δ*exsA* with expression of *exsA* remaining off or induced by overnight growth with rhamnose (2 %). Data pooled from 3 eyes per strain. Error bars show the median with interquartile range. **** P < 0.0001 (Kolmogorov-Smirnov test).
